# Can Serum and Saliva Inflammatory Cytokines Be Considered a Reliable Marker in Chronic Oral Graft-Versus-Host Disease Patients?

**DOI:** 10.3390/jpm14121122

**Published:** 2024-11-27

**Authors:** Giorgia Pugliese, Letizia Nitro, Fabiana Allevi, Federico Biglioli, Matilde Coccapani, Giovanni Felisati, Francesco Ferella, Giorgio Ghilardi, Linda Montavoci, Anna Caretti, Alberto Maria Saibene

**Affiliations:** 1 Otolaryngology Unit, Santi Paolo e Carlo Hospital, Department of Health Sciences, Università degli Studi di Milano, 20142 Milan, Italy; giorgia.pugliese@unimi.it (G.P.); letizia.nitro@unimi.it (L.N.); matilde.coccapani@unimi.it (M.C.); giovanni.felisati@unimi.it (G.F.); francesco.ferella@unimi.it (F.F.); 2 Maxillofacial Surgery Unit, Santi Paolo e Carlo Hospital, Department of Health Sciences, Università degli Studi di Milano, 20142 Milan, Italy; fabiana.allevi@unimi.it (F.A.); federico.biglioli@unimi.it (F.B.); 3 General Surgery Unit, Department of Health Sciences, Università Degli Studi di Milano, 20142 Milan, Italy; giorgio.ghilardi@unimi.it; 4 Biochemistry Laboratory, Department of Health Sciences, Università degli Studi di Milano, San Paolo, 20142 Milan, Italy; linda.montavoci@unimi.it (L.M.); anna.caretti@unimi.it (A.C.)

**Keywords:** cytokines, disease marker, graft-versus-host disease, oral lichen planus, oral disease, oral premalignant disease

## Abstract

**Background/Objectives:** Chronic graft-versus-host disease (cGVHD) and oral lichen planus (LPO) are chronic inflammatory conditions with similar oral manifestations. This study aimed to assess whether serum and salivary cytokines (IL-1α, IL-6, IL-17) could serve as reliable biomarkers for cGVHD. **Methods:** A prospective cohort study was conducted involving cGVHD patients, LPO patients, and healthy controls. Cytokine levels in serum and saliva were measured by ELISA and compared across the groups using the Kruskal–Wallis test. **Results:** IL-17 levels were significantly elevated in the serum of cGVHD patients compared to LPO patients and controls (*p* < 0.05). However, IL-6 and IL-1α did not show significant differences among the groups. A comparison of salivary samples between the three groups did not reach statistical significance (*p* > 0.05). **Conclusions:** This study suggests that IL-17 could be a potential biomarker for cGVHD-related inflammation, warranting further investigation. Salivary samples do not seem to be a reliable biological marker for the diagnosis of cGVHD. The findings underline the need for larger studies to validate these preliminary results.

## 1. Introduction

Graft-versus-host disease (GVHD) is a systemic disorder representing one of the most severe complications of allogeneic hematopoietic stem cell transplantation (allo-HSCT), consisting of immune response-induced tissue damage directed from the graft’s cells towards the host [1,2]. Its incidence has increased in the last decades after the spread of partially compatible HLA transplantation performed through pre-transplant conditioning medications, thanks to improved overall survival of treated patients. It exists as an acute form (acute GVHD, or aGVHD), which typically onsets within 100 days after the HSCT affects the skin, liver, and gastrointestinal system and is a chronic form [3]. Chronic GVHD (cGVHD) onsets after 100 days from the HSCT and can affect the vast majority of the host’s tissues, though the most involved sites are the oral cavity, oropharynx, salivary, and lacrimal glands [4].

Oral manifestations of cGVHD—mucositis, ulcerations, leukoplakia, and erythroplakia—are the most frequent, with a prevalence ranging between 45% and 83% of patients affected [5,6] and impose a significant burden on patients’ health and quality of life. Furthermore, they carry an intrinsic neoplastic potential, with up to 15% of patients being at risk of developing de novo head-and-neck squamocellular cancer, particularly in the oral cavity [7,8].

Graft-versus-host disease (GVHD) is part of a large group of conditions known as Interface Cell-Mediated Mucositis (ICMM), including, among others, oral lichen planus (LPO) [9].

ICMM share a common pathogenic process and morphology of the resulting lesions (imprecisely referred to as lichenoid lesions) [10]. The pathogenesis is based on damage to the cells of the basal layer of the epidermis or mucosa mediated by T lymphocytes, with TNF-α and IFN-γ being the most important mediators in activating and amplifying T lymphocytes’ cytotoxic activity.

As in other inflammatory chronic diseases, it is ascertained that inflammatory cytokines play a role in cGVHD physiopathology [6,11,12,13,14]. Recent studies have demonstrated an increased level of proinflammatory cytokines, particularly interleukin 1 alpha (IL-1α) and interleukin 6 (IL-6), in cGVHD patients’ salivary and blood samples [8,15,16,17]. Despite that, the potential role of inflammatory cytokines as a marker of cGVHD has been little explored [15,18,19,20].

Analyzing cytokines salivary levels in patients affected by this condition and eventually establishing their role as a reliable biomarker of the disease could represent a novel laboratory parameter for patients’ follow-up monitoring. Furthermore, since it is well established the neoplastic potential of these lesions, monitoring cytokines levels could be used to diagnose potential cancerogenesis at a preclinical stage [7,8,15]. Last, little is known about the relationship between salivary and plasma concentrations of these molecules. Therefore, we developed a preliminary prospective evaluation comparing a group of cGVHD with oral involvement with LPO patients and patients free from oral cavity disease.

The aim of the study is to analyze the different inflammatory cytokines levels (IL-1α, IL-6, and IL-17) in two chronic inflammation models (cGVHD and LPO), which share similar inflammatory patterns and clinical oral manifestations. Comparing cytokines levels in saliva and serum could allow evaluation of the reliability of salivary samples, a much more accessible and less invasive procedure.

## 2. Materials and Methods

This study was designed as a prospective observational cohort study. The minimum sample size was determined in 8 cGVHD patients and 8 healthy controls based on literature data concerning healthy controls and patients affected by systemic conditions considered at risk for oral carcinoma (salivary IL-6 0.002 ± 0.002 pg/mL and 0.431 ± 0.217 pg/mL in cGVHD patients and healthy controls, respectively [21,22]). Alpha and beta errors were set as 0.01 and 0.2, respectively. A similarly sized population of LPO patients was deemed, therefore, acceptable.

The cGVHD group (Group A) included all consecutive adult patients admitted for the first time between April 2023 and March 2024 at our second-level oropharyngeal disease clinic with a known diagnosis of cGVHD following HSCT. They did not receive topical or systemic corticosteroid therapy in the two prior months. The LPO control group (Group B) included patients with a clinical and histological diagnosis of LPO attending the same second-level oropharyngeal clinic who did not receive topical or systemic corticosteroid therapy in the two prior months. The healthy control group (Group C) was represented by patients attending our clinic for reasons other than oral disease and free from any oral disease upon examination. They voluntarily participated in the study.

All patients underwent a salivary and venous sampling at the evaluation. Demographic information was collected for all groups, including sex, age, smoking, alcohol consumption, substance use, systemic, dermatological, and oral chronic diseases, and recent (up to 12 months before evaluation) dental treatments.

All the samples collected were stored at −80 °C until cytokine dosage. Quantification of IL-1α, IL-6, and IL-17 was performed by biomarker multiplex immunoassays on the Luminex^®^ Platform (Thermo Fisher Scientific, Waltham, MA, USA). Every determination has been performed in duplicate. Data were analyzed in order to obtain descriptive statistics.

Due to the reduced sample size, all data except binomial variables were considered non-parametric data. Therefore, we used the median, interquartile range (IQR), and minimum–maximum range (MMR) as descriptive statistics for continuous data, which are indeed reported as median ± IGR (MMR) unless otherwise stated. Median values of plasma and saliva cytokines were compared between groups with a Kruskal–Wallis test. A Bonferroni a posteriori statistical correction was applied for these tests. Correlation between different cytokines values was performed with a Spearman test. All statistical tests were performed using SPSS v. 28 (IBM Corp., Armonk, NY, USA).

## 3. Results

Ten patients were recruited for Group A during the study period; therefore, as many patients were recruited for groups B and C. The population consisted of a total of 19 females and 11 males, with a median age of 63 years (IQR 48.75–70.5). Demographics and clinical data regarding the three groups are reported in Table 1. All patients denied substance use.

The concentration values of the cytokines in serum and saliva are reported in pg/mL, as shown in Table 2 and Table 3, respectively.

IL-6 median concentration in all patients’ serum is 0.39 (IQR 0.2375–0.79). Median concentration is 0.47 (IQR 0.395–0.65) in Group A, 0.245 (IQR 0.2075–0.3825) in Group B, and 1.23 (0.26–12.265) in Group C. IL-17 median concentration in all groups is equal to 0.55 (IQR 0–1.1). The median concentration in Group A is 1.1 (IQR 1.1–1.1), in Group B is 0.55 (IQR 0–1.1), and in Group C is 0 (0–0).

The median serum concentration of IL-1α is equal to 0 (IQR 0–0) considering all groups (as a whole and individually).

Regarding salivary samples, the IL-6 median concentration in the total of 30 patients is equal to 3.28 (IQR 1.8875–9.975). Median concentration is 6.62 (IQR 1.7675–16.9475) in Group A, 0.12 (IQR 0–1.84) in Group B, and 527.36 (IQR 182.83–1000) in Group C.

IL-17 median salivary concentration for all patients is 0 (IQR 0–1.02). The median concentration is 0.12 (0–1.84) in Group A, 0 (IQR 0–0) in Group B, and 0.12 (IQR 0–1.02) in Group C.

For IL-1α, the salivary median concentration of all patients is 620.6 (IQR 284.08–1000). Group A median is 527.36 (IQR 182.83–1000), Group B median is 529.03 (IQR 392.87–1000), and Group C median is 1000 (IQR 203.2375–1000).

The comparison of cytokine concentration between the three groups was performed through the Kruskal–Wallis test. Serum IL-17 level is higher in Group A compared to Groups B and C, with a *p*-value < 0.05. Serum IL-6 level is higher in Group A, but the difference is not statistically significant (*p*-value > 0.05). Serum IL-1α level was unchanged as well (*p*-value > 0.05).

The comparison between salivary samples between the three groups did not reach statistical significance, with the *p*-value > 0.05 (details in Table 4).

Statistical significance is still observed between cGVHD patients and controls (groups A and C) after performing the Bonferroni adjustment a posteriori. Instead, it is not significant the comparison between cGVHD and LPO patients (groups A and B) and between LPO patients and controls (groups B and C).

Spearman’s test did not show any relevant correlation between cytokines’ values.

## 4. Discussion

Oral manifestations of cGVHD significantly affect patients’ health and quality of life, requiring close monitoring of lesions and symptoms. Moreover, given the increased risk (up to 15%) of developing a de novo head-and-neck squamocellular cancer (SCC) [7,8], management of cGVHD patients should be based on a long-term follow-up in order to early detect malign progression. Clinical management is based on oral cavity examination, upper airway endoscopy (with the adjuvance of Narrow Band Imaging, NBI), and eventually, biopsy, which remains the gold standard for identifying neoplastic lesions. There is currently no way to define the early risk of developing more or less severe symptoms or to determine their potential neoplastic evolution. However, given some scientific evidence of the correlation between levels of systemic and local inflammation and the development of SCC, it is possible that monitoring the concentration of various inflammatory mediators in the biological fluids of these patients could provide useful information for clinical stratification and neoplastic risk and could help in monitoring the response to therapy.

This preliminary study was conducted to explore cytokine levels in blood and saliva among patients with cGVHD and LPO, aiming to determine whether these inflammatory markers can serve as reliable indicators of disease activity. Although the literature supports the use of cytokines as markers of inflammation, specific data regarding cGVHD are still limited [20].

We chose to focus on IL-1α, IL-6, and IL-17 due to their known role in systemic and local inflammation within the context of GVHD. Our study analyzed these cytokines in serum and saliva between three groups: cGVHD patients not receiving corticosteroids, LPO patients with well-controlled symptoms without therapy, and healthy volunteers. We expected to observe distinct cytokine concentration patterns reflective of varying systemic inflammation levels among the groups. Furthermore, we chose to analyze the role of IL-1α and IL-6 due to their known implication in head and neck squamous cell carcinoma development, while IL-17 was chosen due to its known role in fibrotic changes, which often characterize oral lesions from both GVHD and LPO.

Our results indicated that IL-6, a key cytokine in the inflammatory response underlying GVHD, did not significantly accumulate in cGVHD patients compared to controls, either in serum or saliva. This finding diverges from existing literature, likely due to the controlled therapeutic state of our cGVHD cohort [15,23,24]. It should still be accounted for that serum IL-6 levels tended to be higher (without reaching statistical significance) in Group A compared to the other two groups after performing the Kruskal–Wallis test.

The concentration of IL-1α, widely studied for its role in autoimmune diseases, was unchanged in cGVHD and in the other groups, despite previous findings of elevated IL-1α in murine models and in saliva samples of cGVHD patients [25,26,27]. This lack of significance might reflect the small sample size or the need for more refined measurement techniques.

Conversely, what emerges from our analysis is that IL-17 increased serum level in cGVHD patients with oral manifestations is statistically significant, compared to both patients affected by LPO and healthy patients (controls).

This finding suggests that IL-17, beyond the traditionally studied cytokines like IL-6, may play a critical role in the pathogenesis of cGVHD in accordance with its established function in autoimmune and autoinflammatory responses [28,29,30]. IL-17’s ability to recruit neutrophils and stimulate the production of antimicrobial substances highlights its importance in maintaining mucosal integrity and its potential involvement in the inflammatory cascade of GVHD [25,26,27].

The role of Th17 lymphocytes, which primarily produce IL-17, further underscores the cytokine’s significance. These cells, central to mucosal immunity, may contribute to GVHD pathology through mechanisms that are not yet fully understood. Animal studies have shown that donor-derived Th17 cells can either exacerbate or mitigate GVHD symptoms depending on the model, suggesting a complex interaction between these cells and the disease process [31,32,33,34,35]. The observed increase in IL-17 in our cGVHD patients supports the hypothesis that IL-17 and Th17 cells play a key role in the disease’s progression [36,37].

Though our results are interesting from a speculative point of view, one of the major limitations of our work applies to our initial aims, i.e., to test whether cytokines levels in saliva and serum are reliable tools for cGVHD diagnosis. Though the small size of our sample might have hampered the conclusions, what emerged from our analysis is that salivary samples do not seem to be a reliable marker in diagnosing cGVHD, as no significant difference has been observed in salivary cytokine’s levels among the three groups.

Another major limitation of our work is the focus on patients with cGVHD, a limitation that is intrinsic to our patient population, as patients with acute GVHD are followed more intensively in other departments. This means that our study did not have the opportunity to widen its considerations on more cytokines such as IFN-γ, G-CSF, GM-CSF, IL-2, IL-3, IL-5, and IL-13, which all have a known role in acute GVHD [12]. Exploring the role of these cytokines in acute and chronic GVHD and in other oral diseases such as LPO might provide further interesting insight.

While this study provides valuable insights into cytokine behavior in cGVHD, it is not without limitations. The small sample size limits the application of our findings on a larger scale, and the cross-sectional nature of the study does not allow for the observation of cytokine level changes over time or in response to treatment. Further research should focus on larger cohorts and longitudinal studies to better understand the role of these cytokines in cGVHD and to assess their potential as biomarkers for disease progression and therapeutic response.

## 5. Conclusions

In conclusion, this study highlights the importance of IL-17 as a potential marker for cGVHD-related inflammation and suggests that further research into the Th17/IL-17 axis could provide new avenues for monitoring and treating this complex condition. Our findings, taking into account the sample size’s limitation, reveal that salivary samples do not seem to be a reliable biological marker for the diagnosis of cGVHD. Despite the small cohort, our findings are consistent with the literature and offer a promising foundation for future investigations into the role of cytokines in cGVHD and other chronic inflammatory diseases.

## Figures and Tables

**Table 1:** Demographic and clinical data.

Group	M:F	Age (Mean)	Smoke	Alcohol	Comorbidities	Oral Pathologies	Skin Pathologies	Dental Procedures (Last Year)
A	4:6	60.3	5 p	2 p	4 p	0	1 p	1 p
B	2:8	68.7	6 p	2 p	7 p	0 (rather than LPO)	1 p	4 p
C	5:5	50.7	5 p	5 p	4 p	0	1 p	0

**Table 2:** IL-6, IL-17, and IL-1α serum levels in Groups A, B and C.

Group	Patient	IL-6	IL-17	IL-1α
A	1	0.36	1.1	0
	17	0.39	1.1	0
	19	0.41	1.1	0
	2	0.18	1.1	0
	20	0.45	1.1	0
	22	0.7	1.1	0
	23	0.82	1.1	0
	30	0.49	1.1	0
	8	0.9	1.1	0
	9	0.5	1.1	0
	Median (IQR)	0.47 (0.395–0.65)	1.1 (0–1.1)	0 (0–0)
B	10	0.04	1.1	0
	11	0.2	1.1	0
	14	0.36	1.1	0
	15	0.23	1.1	0
	16	0.19	1.43	0
	18	0.23	0	0
	21	1.02	0	0
	24	0.52	0	0
	25	0.26	0	0
	26	0.39	0	0
	Median (IQR)	0.245 (0.2075–0.3825)	0.55 (0.2075–0.3825)	0 (0.26–12.265)
C	12	224.16	0	0
	13	261.25	0	0
	27	8.92	0	2.39
	28	13.38	0	0
	29	2.1	0	0
	3	0.21	0	0
	4	0.21	0	0
	5	0.26	0	0
	6	0.26	0	0
	7	0.36	0	0
	Median (IQR)	1.23 (0.26–12.265)	0 (0–0)	0 (0–0)
A + B + C	Median	0.39	0.55	0
	IQR	0.2375–0.79	0–1.1	0–0

**Table 3:** IL-6, IL-17, and IL-1α salivary levels in Groups A, B, and C.

Group	Patient	IL-6	IL-17	IL-1α
A	1	19.09	0	1000
	17	2	0.12	1000
	19	10.52	2.64	527.36
	2	22.09	0	1000
	20	2.96	1.84	343.37
	22	257.87	3.03	110.16
	23	10.28	1.02	182.83
	30	0.25	0	10.77
	8	1.69	0	712.17
	9	1.04		
	Median (IQR)	6.62 (1.7675–16.9475)	0.12 (0–1.84)	527.36 (182.83–1000)
B	10	0.68	0	1000
	11	2.44	0	996.26
	14	0.51	0	379.94
	15	5.36	7.36	1000
	16	3.33	0	180.01
	18	3.13	0	1000
	21	52.94		
	24	3.4	0	392.87
	25	8.68	0	503.35
	26	3.65	0	529.03
	Median (IQR)	3.365 (2.6125–4.9325)	0 (0–0)	529.03 (392.87–1000)
C	12	1.85	0	1000
	13	2.29	0	73.99
	27	9.06	1.02	1000
	28	0.77	0	54.15
	29	7.97	3.03	165.04
	3	0.58	2.64	317.83
	4	33.71	0.12	1000
	5	3.23	1.02	1000
	6	2.94	0	1000
	7	55.18	0.12	1000
	Median (IQR)	3.085 (1.96–8.7875)	0.12 (0–1.02)	1000 (203.2375–1000)
A + B + C	Median	3.28	0	620.6
	IQR	1.8875–9.975	0–1.02	284.08–1000

**Table 4:** Statistical analysis (Kruskal–Wallis test) of salivary and serum cytokines’ concentration in Groups A, B, and C.

Cytokine (Saliva)	*p*-Value	Cytokine (Serum)	*p*-Value
IL-6	0.924	IL-6	0.108
IL-17	0.169	IL-17	<0.001 (after Bonferroni a posteriori correction, inter-group comparison *p* values were A–C, *p* = 0; A–B 0.182; and B–C 0.063)
IL-1α	0.773	IL-1α	0.368

## Data Availability

All data pertaining to this article are available from the corresponding author upon reasonable request.
